# Risk factors for deep surgical site infection after posterior cervical spine surgery in adults: a multicentre observational cohort study

**DOI:** 10.1038/s41598-021-87110-4

**Published:** 2021-04-06

**Authors:** Satoshi Ogihara, Takashi Yamazaki, Michio Shiibashi, Hirotaka Chikuda, Toru Maruyama, Kota Miyoshi, Hirohiko Inanami, Yasushi Oshima, Seiichi Azuma, Naohiro Kawamura, Kiyofumi Yamakawa, Nobuhiro Hara, Jiro Morii, Rentaro Okazaki, Yujiro Takeshita, Junji Nishimoto, Sakae Tanaka, Kazuo Saita

**Affiliations:** 1 Department of Orthopaedic Surgery, Saitama Medical Center, Saitama Medical University, 1981 Kamoda, Kawagoe, Saitama 350-8550 Japan; 2 Department of Orthopaedic Surgery, Musashino Red Cross Hospital, 1-26-1 Kyonancho, Musashino, Tokyo 180-8610 Japan; 3 Information Technology Center, Saitama Medical University, 38 Morohongo Moroyama-machi, Iruma-gun, Saitama 350-0495 Japan; 4 Department of Orthopaedic Surgery, Gunma University Graduate School of Medicine, 3-39-22 Showa, Maebashi, Gunma 371-8511 Japan; 5 Department of Orthopaedic Surgery, Saitama Rehabilitation Center, 148-1 Nishikaizuka, Ageo, Saitama 362-0057 Japan; 6 Department of Orthopaedic Surgery, Yokohama Rosai Hospital, 3211 Kozukuecho, Kouhoku-ku, Yokohama, Kanagawa 222-0036 Japan; 7 Department of Orthopaedic Surgery, Inanami Spine and Joint Hospital, 3-17-5 Higashishinagawa, Shinagawa-ku, Tokyo 140-0002 Japan; 8 Department of Orthopaedic Surgery, Faculty of Medicine, University of Tokyo, 7-3-1 Hongo, Bunkyo-ku, Tokyo 113-8655 Japan; 9 Department of Orthopaedic Surgery, Saitama Red Cross Hospital, 1-5 Shintoshin, Chuo-ku, Saitama 330-8553 Japan; 10 Department of Spine and Orthopaedic Surgery, Japanese Red Cross Medical Center, 4-1-22 Hiroo, Shibuya-ku, Tokyo 150-8935 Japan; 11 Department of Orthopaedic Surgery and Musculoskeletal Oncology, Tokyo Metropolitan Komagome Hospital, 3-18 Honkomagome, Bunkyo-ku, Tokyo 113-0021 Japan; 12 Department of Orthopaedic Surgery, Sanraku Hospital, 2-5 Surugadai, Kanda, Chiyoda-ku, Tokyo 101-8326 Japan; 13 Department of Rehabilitation, Saitama Medical Center, Saitama Medical University, 1981 Kamoda, Kawagoe, Saitama 350-8550 Japan

**Keywords:** Microbiology, Diseases, Health care, Risk factors

## Abstract

Surgical site infection (SSI) is a serious complication following spine surgery and is correlated with significant morbidities, poor clinical outcomes, and increased healthcare costs. Accurately identifying risk factors can help develop strategies to reduce this devastating consequence; however, few multicentre studies have investigated risk factors for SSI following posterior cervical spine surgeries. Between July 2010 and June 2015, we performed an observational cohort study on deep SSI in adult patients who underwent posterior cervical spine surgery at 10 research hospitals. Detailed patient- and procedure-specific potential risk variables were prospectively recorded using a standardised data collection chart and were reviewed retrospectively. Among the 2184 consecutive adult patients enrolled, 28 (1.3%) developed postoperative deep SSI. Multivariable regression analysis revealed 2 statistically significant independent risk factors: occipitocervical surgery (*P* < 0.001) and male sex (*P* = 0.024). Subgroup analysis demonstrated that occipitocervical surgery (*P* = 0.001) was the sole independent risk factor for deep SSI in patients with instrumented fusion. Occipitocervical surgery is a relatively rare procedure; therefore, our findings were based on a large cohort acquired using a multicentre study. To the best of our knowledge, this is the first study to identify occipitocervical procedure as an independent risk variable for deep SSI after spinal surgery.

## Introduction

Surgical site infection (SSI) after spine surgery is one of the most notable postoperative complications; it leads to higher morbidity, mortality, and healthcare costs and is associated with longer hospital stays and poorer patient outcomes. The incidence of SSI in the spine is reportedly between 0.65 and 12% and varies according to the type of surgery and target population^1–3^. The accurate identification of risk factors for SSI can serve as a theoretical basis for promoting preventive measures to guard against this devastating complication.

Numerous studies have sought to identify the risk variables regarding SSI following spine surgery to develop strategies to decrease its incidence. Several estimated risk variables have been reported in previous research, such as advanced age^4^, male sex^5–7^, high body mass index (BMI)^4,8–11^, diabetes^4,9,11,12^, revision status^4^, smoking^4^, high American Society of Anesthesiologists (ASA) score^8^, chronic corticosteroid use^6,13,14^, spinal instrumentation^8^, posterior surgical approach^2,8^, spinal trauma^6,12^, tumour resection^2^, rheumatoid arthritis^15^, dural tear^11^, increased intraoperative bleeding^5,11,16^, and prolonged operative duration^5,8,14,16,17^. However, many of these studies were performed retrospectively at individual institutions and were limited by their relatively small sample size. Even large-sample studies using nationwide databases were found to be inadequate for examining individual operative procedures in detail^3^. Regarding cervical spinal surgeries in particular, there is a lack of data from multicentre studies that have gathered clinical data prospectively concerning SSI risk factors.

High-quality studies that use a multicentre design with a large cohort and examine a wide range of potential risk factors are still required to determine independent risk variables for SSI after cervical spine surgery. Multivariable analysis should be used to adjust for the concurrence of multiple correlated variables among individual patients. Therefore, the purpose of the current research was to investigate the incidence of deep SSI development after posterior cervical spinal surgery in adults, and to identify the independent risk factors thereof, via a multicentre observational cohort study utilising prospectively gathered data from a registry of over 2000 patients.

## Methods

### Study design and selection criteria

This observational cohort study aimed at investigating deep SSI after posterior cervical spine surgery that was performed in adult patients between July 1, 2010 and June 30, 2015 at 10 research hospitals in Japan. Each patient had undergone follow-up for a minimum of 12 months; detailed patient-specific and procedure-specific potential risk variables were recorded prospectively utilising a standardised data collection chart. Patients who underwent surgery for the treatment of spinal infection were excluded from the analysis. We also excluded patients aged < 18 years as well as those who underwent posterior instrumentation removal, endoscopic surgery, or single-stage anterior–posterior surgery to ensure homogeneity of the study group. This study was carried out in accordance with the Declaration of Helsinki, and the study protocol was approved by the institutional review boards of Saitama Medical University, Sagamihara National Hospital, Musashino Red Cross Hospital, Japanese Red Cross Medical Center, the University of Tokyo, Tokyo Metropolitan Komagome Hospital, Yokohama Rosai Hospital, Iwai Orthopaedic Medical Hospital, Saitama Red Cross Hospital, and Sanraku Hospital. The requirement for informed consent was waived by each study hospital owing to the observational nature of this study, and opt-out information was posted on the website of Saitama Medical University. All clinical data were anonymised and de-identified before analysis.

### Data collection

Data regarding patient-specific and procedure-specific variables were prospectively gathered from the medical records using a standardised data collection chart. The patient-specific variables included sex, age at the time of surgery, height, weight, BMI, diabetes mellitus, smoking, ASA score^18^, preoperative chronic steroid administration, haemodialysis, surgical pathology (degenerative diseases, spinal trauma, spinal tumour, or rheumatoid arthritis), and previous surgery. Additionally, procedure-specific possible risk variables for SSI were assembled and analysed; these included operative duration, intraoperative bleeding, spinal surgical level (occipital, cervical, and/or thoracic), use of spinal instrumentation, emergency surgery, dural tear, iliac crest bone grafting, use of a surgical microscope, use of intraoperative fluoroscopy, use of a bio-clean room, intravenously administered prophylactic antibiotics, intrawound administration of powdered vancomycin, and type of hospital (academic or non-academic). The study was conducted in an observational manner without a pre-arranged SSI prophylactic intervention protocol. Accordingly, there was no preoperative standardized protocol regarding either preoperative smoking cessation; preoperative control of diabetes aiming at the reduction of blood sugar or serum HbA1c to a certain level; or methicillin-resistant *Staphylococcus aureus* (MRSA) preoperative colonization test.

Patients were diagnosed with deep wound infections at the participating hospitals based on the Centres for Disease Control and Prevention’s criteria of SSI^19^. The results of microbiological cultures from all patients who developed deep SSI were also recorded. For patients with deep SSI who underwent open debridement, microbiological cultures were obtained to confirm the occurrence of SSI and determine further treatment.

### Statistical analysis

The correlations between deep SSI and the potential risk variables were analysed. Univariate analysis used Student’s t-test for continuous variables, and Fisher’s exact test to compare categorical variables. On univariate analysis, significant factors and those associated with deep SSI (*P* < 0.20) were incorporated into a stepwise multivariable logistic regression model to identify independent risk factors for deep SSI. Statistical analyses were executed using SPSS Statistics ver. 24 (IBM Corp., Armonk, NY). *P* < 0.05 was considered statistically significant. Regarding the calculation of the statistical power, we performed a post hoc power analysis using G*Power 3.1.9.7^20^.

## Results

A total of 2,184 consecutive patients (682 women and 1,502 men with a mean age of 65.9 years [range, 18–93 years]) from 10 Japanese hospitals were enrolled between July 2010 and June 2015; their demographic characteristics are shown in Table 1. The overall incidence of postoperative deep SSI was 1.3% (28 patients).

**Table 1:** Demographic characteristics of the deep SSI and non-deep SSI groups in the entire cohort.

Characteristic	Deep SSI group (n = 28)	Non-deep SSI group (n = 2156)	*P* value^a^
Age (years), mean ± SD	64.1 ± 15.0	65.9 ± 12.4	0.441
Male sex, n (%)	23 (82.1)	1479 (68.6)	0.087
Body mass index (kg/m^2^), mean ± SD	24.3 ± 3.5	23.7 ± 3.8	0.399
Surgical pathology			
Degenerative diseases, n (%)	21 (75.0)	1825 (84.6)	0.129
Spinal trauma, n (%)	3 (10.7)	156 (7.2)	0.332
Spinal tumour, n (%)	2 (7.1)	90 (4.2)	0.334
Rheumatoid arthritis, n (%)	2 (7.1)	55 (2.6)	0.165
ASA score ≥ 2, n (%)	26 (92.9)	1777 (82.4)	0.109
Diabetes mellitus, n (%)	7 (25.0)	452 (17.0)	0.190
Diabetes mellitus with insulin use, n (%)	3 (10.7)	452 (5.0)	0.164
Haemodialysis, n (%)	1 (3.6)	116 (5.4)	0.552
Smoking, n (%)	6(21.4)	299 (13.9)	0.187
Preoperative steroid therapy, n (%)	2 (7.1)	109 (5.1)	0.421
Spinal surgical level			
Operation including occipital bone, n (%)	9 (32.1)	91 (4.2)	< 0.001
Operation including thoracic spine, n (%)	5 (17.9)	121 (5.6)	0.020
Posterior instrumentation, n (%)	12 (42.9)	426 (19.8)	0.005
Revision surgery, n (%)	3 (10.7)	132(6.1)	0.248
Iliac bone grafting, n (%)	8 (28.6)	142(6.6)	< 0.001
Dural tear, n (%)	0 (0.0)	118 (5.5)	0.209
Use of an operative microscope, n (%)	0 (0.0)	59 (2.7)	0.462
Use of an intraoperative fluoroscopy	11 (39.3)	286 (11.3)	0.001
Use of a bio-clean room, n (%)	5 (17.6)	678 (31.4)	0.087
Emergency surgery, n (%)	1 (3.6)	158(7.3)	0.383
Operative time (min), mean ± SD	204.9 ± 116.1	163.9 ± 76.7	0.073
Intraoperative bleeding (ml), mean ± SD	279.9 ± 238.6	276.3 ± 352.8	0.958
Prophylactic intravenous administration of CEZ, n (%)	25 (89.3)	2060 (95.5)	0.131
Powdered vancomycin administration to the operative wound, n (%)	0 (0.0)	134 (6.2)	0.168
Surgery at academic hospitals	9 (32.1)	500 (23.2)	0.185

^a^Fisher’s exact test was used for categorical variables, while Student’s t-test was used for continuous variables. *SSI* surgical site infection, *SD* standard deviation, *ASA* American Society of Anesthesiologists, *CEZ* cefazolin.

The relationships between investigated variables and deep SSI are provided in Table 1. Univariate analysis identified several significant risk variables for SSI including occipital bone involvement (decompression and/or instrumentation) during surgery, thoracic spine involvement (decompression and/or instrumentation) during surgery, posterior instrumentation, use of intraoperative fluoroscopy, and iliac bone grafting. Factors that were significant on univariate analysis as well as those with *P* values < 0.20 (male sex, ASA score ≥ 2, diabetes mellitus with insulin use, use of a bio-clean room, prophylactic intravenous administration of cefazolin, and operative time) were subjected to multivariable analysis for further examination of the risk variables for deep SSI. The final multivariable model (Table 2) shows two independent risk factors for deep SSI after adjusting for other risk variables. According to these logistic regression models, involvement of the occipital bone during surgery was found to be strongly correlated with an increased risk of deep SSI (odds ratio [OR], 14.26; 95% confidence interval [CI], 6.07–33.52; *P* < 0.001). In our cohort, 95 patients received instrumented fusion out of 100 patients who underwent surgery that involved the occipital bone, and all 9 patients who developed deep SSI after surgeries involving the occipital bone received instrumented fusion. The present analysis also revealed that men had a 3.21-fold higher risk of deep SSI than women (95% CI, 1.17–8.86; *P* = 0.024). Regarding the post hoc statistical power analysis, the results regarding the surgical involvement of the occipital bone had effect size w = 0.606 and power = 1.000, while the results regarding the male sex had effect size w = 0.292 and power = 1.000. Therefore, it was considered that the results of the present study had sufficient statistical power.

**Table 2:** Multivariate logistic regression analysis of factors associated with deep SSI after posterior cervical spinal surgery in the entire cohort.

Characteristic	OR (95% CI)	*P* value
Male sex	3.21 (1.17–8.86)	0.024
Degenerative diseases		0.892
Rheumatoid arthritis		0.941
ASA score ≥ 2		0.171
Diabetes mellitus with insulin use		0.087
Smoking		0.201
Operation including occipital bone	14.26 (6.07–33.52)	< 0.001
Operation including thoracic spine		0.055
Posterior instrumentation		0.765
Iliac bone grafting		0.429
Use of an intraoperative fluoroscopy		0.081
Use of a bio-clean room		0.143
Operative time (min)		0.689
Prophylactic intravenous administration of CEZ		0.141
Powdered vancomycin administration to the operative wound		0.078
Surgery at academic hospitals		0.769

*SSI* surgical site infection, *OR* odds ratio, *CI* confidence interval, *ASA* American Society of Anesthesiologists, *CEZ* cefazolin.

### Subgroup analysis of patients with instrumented fusion and non-fusion surgery

In total, 438 patients in the cohort underwent fusion surgery with posterior instrumentation, among whom 12 (2.7%) had SSIs (Table 1). Moreover, 1746 patients had non-fusion surgery, among whom 16 (0.92%) had SSIs. The difference in the occurrence of deep SSI between these 2 groups was significant (*P* = 0.005) (Table 1).

In the posterior instrumentation fusion cohort, 9 variables (surgery that involved the occipital bone [*P* < 0.001], use of intraoperative fluoroscopy [*P* = 0.009], iliac bone grafting [*P* = 0.013], powdered vancomycin administration into the surgical wound [*P* = 0.070], surgery at academic hospitals [*P* = 0.073], use of a bio-clean room [*P* = 0.076], emergency surgery [*P* = 0.120], revision surgery [*P* = 0.189], and surgery that involved the thoracic spine [*P* = 0.193]) were correlated with deep SSI on univariate analysis (*P* < 0.2) (Table 3). A multivariate stepwise regression model that incorporated these 9 variables showed that surgery that involved the occipital bone (*P* = 0.001) was the sole independent risk variable for deep SSI in patients with instrumented fusion surgery (Table 4). Regarding the post hoc statistical power analysis, the results of the surgical involvement of the occipital bone had an effect size w = 1.266 and power = 1.000. Therefore, it was considered that the results of the current analysis had sufficient statistical power.

**Table 3:** Demographic characteristics of the deep SSI and non-deep SSI groups in the instrumented fusion cohort.

Characteristic	Deep SSI group (n = 12)	Non-deep SSI group (n = 426)	*P* value^a^
Age (years), mean ± SD	62.7 ± 16.7	64.6 ± 13.7	0.641
Male sex, n (%)	8 (66.7)	249 (58.5)	0.39
Body mass index (kg/m^2^), mean ± SD	23.7 ± 3.0	22.8 ± 3.8	0.403
Preoperative diagnosis			
Degenerative diseases, n (%)	5 (41.7)	200 (46.9)	0.475
Spinal trauma, n (%)	3 (25.0)	123 (28.9)	0.530
Spinal tumour, n (%)	2 (16.7)	48 (11.3)	0.407
Rheumatoid arthritis, n (%)	2 (16.7)	47 (11.0)	0.396
ASA score ≥ 2, n (%)	12 (100.0)	373 (87.6)	0.208
Diabetes mellitus, n (%)	2 (16.7)	55 (12.9)	0.478
Diabetes mellitus with insulin use, n (%)	1 (8.3)	16 (3.8)	0.382
Haemodialysis, n (%)	0 (0.0)	29 (2.8)	0.435
Smoking, n (%)	2 (16.7)	36 (8.5)	0.280
Preoperative steroid therapy, n (%)	2 (16.7)	53 (12.4)	0.458
Spinal surgical level			
Operation including occipital bone, n (%)	9 (75.0)	86 (20.2)	< 0.001
Operation including thoracic spine, n (%)	4 (33.3)	82 (19.2)	0.193
Revision surgery, n (%)	3 (25.0)	53 (12.4)	0.189
Iliac bone grafting, n (%)	8 (66.7)	131 (30.8)	0.013
Dural tear, n (%)	0 (0.0)	31 (7.3)	0.410
Use of an operative microscope, n (%)	0 (0.0)	5 (1.2)	0.870
Use of an intraoperative fluoroscopy	11 (91.7)	235 (55.2)	0.009
Use of a bio-clean room, n (%)	1 (8.3)	133 (31.2)	0.076
Emergency surgery, n (%)	0 (0.0)	70 (16.4)	0.120
Operative time (min), mean ± SD	239.6 ± 102.4	275.2 ± 145.1	0.242
Intraoperative bleeding (ml), mean ± SD	375.4 ± 287.4	449.8 ± 536.0	0.633
Prophylactic intravenous administration of CEZ, n (%)	10 (83.3)	388 (91.1)	0.301
Powdered vancomycin administration to the operative wound, n (%)	0 (0.0)	86 (20.2)	0.070
Surgery at academic hospitals	6 (50.0)	112 (26.3)	0.073

^a^Fisher’s exact test was used for categorical variables, while Student’s t-test was used for continuous variables. *SSI* surgical site infection, *SD* standard deviation, *ASA* American Society of Anesthesiologists, *CEZ* cefazolin.

**Table 4:** Multivariate logistic regression analysis for deep surgical site infection in the instrumented fusion cohort.

Characteristic	OR (95% CI)	*P* value
Operation including occipital bone	9.90 (2.60–37.74)	0.001
Operation including thoracic spine		0.079
Revision surgery		0.223
Iliac bone grafting		0.893
Use of an intraoperative fluoroscopy		0.071
Use of a bio-clean room		0.250
Emergency surgery		0.223
Powdered vancomycin administration to the operative wound		0.098
Surgery at academic hospitals		0.604

*OR* odds ratio, *CI* confidence interval.

In the non-instrumentation fusion group, 2 variables (male sex [*P* = 0.033] and diabetes [*P* = 0.149]) were correlated (*P* < 0.2) with deep SSI on univariate analysis. A multivariate stepwise regression model incorporating these 2 variables showed that there was no independent risk variable for deep SSI with statistical significance in the non-fusion group (*P* values for male sex and diabetes: 0.080 and 0.153, respectively).

### Microbiological characteristics of SSI

Microbiologic cultures were acquired from all 28 patients with deep SSI, among which 22 (78.6%) were positive; moreover, 27 (96.4%) underwent open debridement. Nineteen of 22 patients with positive cultures (86.4%) had a single organism isolated, while 3 demonstrated polymicrobacterial growth (2 patients had MRSA + *Propionibacterium acnes*, and 1 had coagulase-negative *Staphylococcus* + *Corynebacterium* + *Enterococcus*). *Staphylococcus aureus* was present in 59.1% (13/22) of the positive cultures (including the patient with polymicrobacterial growth), with 30.8% (4/13) of these isolates demonstrating MRSA. Coagulase-negative *Staphylococcus* was the next most common organism, as it was found in 22.7% (5/22) of the positive cultures; methicillin resistance was noted in 60.0% (3/5) of these cultures (Table 5).

**Table 5:** Microbiological characteristics of surgical site infections.

Organism(s)	No. of patients
*Staphylococcus aureus*	9
Methicillin-resistant CNS	3
MRSA	2
MRSA + *Propionibacterium acnes*	2
*Pseudomonas aeruginosa*	2
CNS	1
*Propionibacterium acnes*	1
CNS + *Corynebacterium* + *Enterococcus*	1
*Enterobacter*	1
Unknown	6

*CNS* coagulase-negative staphylococci, *MRSA* methicillin-resistant *Staphylococcus aureus.*

## Discussion

In the current research, we sought independent risk factors for the development of deep SSI in adult patients following posterior cervical spine surgery. The number of published studies to date on SSI development following posterior cervical spinal surgery is small. This may be related to the fact that relatively fewer patients underwent cervical spine surgery than those who underwent lumbar spine surgery^21,22^. Several studies have found that the incidence of SSI after posterior cervical spinal procedures is higher than that observed after anterior approaches^14,23^. To the best of our knowledge, all existing studies on SSI risk factors following posterior cervical spinal surgeries are single-centre investigations^10^ or retrospective studies using nationwide databases^14,17^. Single-centred studies at individual institutes were limited by their relatively small sample sizes and inherent selection biases. In addition, retrospective investigations using nationwide databases, even with large sample sizes, have some disadvantages in terms of clinical data collection, as they are considered to be inadequate for evaluating patient-specific and procedure-specific variables in detail (e.g., revision surgery, dural tear, and surgery involving the occipital bone)^3^. Furthermore, nationwide databases based on administrative claims did not include post-discharge information; therefore, the incidence of SSI was possibly under-reported. To the best of our knowledge, ours is the first study to investigate risk factors for SSI in patients who underwent posterior cervical spinal surgery using a multicentre observational cohort study in which the clinical data was collected prospectively.

The current study revealed that occipitocervical surgery was strongly correlated with the risk of developing deep SSI, especially posterior instrumentation fusion surgery. There are a limited number of reports that provide information on the incidence of SSI after occipitocervical surgery to date, all of which describe retrospective case series at individual institutes^24–27^. The incidence of SSI following occipitocervical instrumented fusion, as reported in previous studies involving over 50 patients, is between 0.4 and 20.3%; the rate varies with the type of surgical procedure, the era of investigation, and studied population^24–27^. In the current research, the incidence of deep SSI after occipitocervical surgery was 9.0% (9 of 100 patients were infected) while the incidence of deep SSI after occipitocervical instrumentation fusion surgery was 9.5% (9 of 95 patients). All the infected patients underwent instrumentation fusion surgery, and our SSI rates were consistent with those described by previous investigators^24–27^.

**Figure 1:**
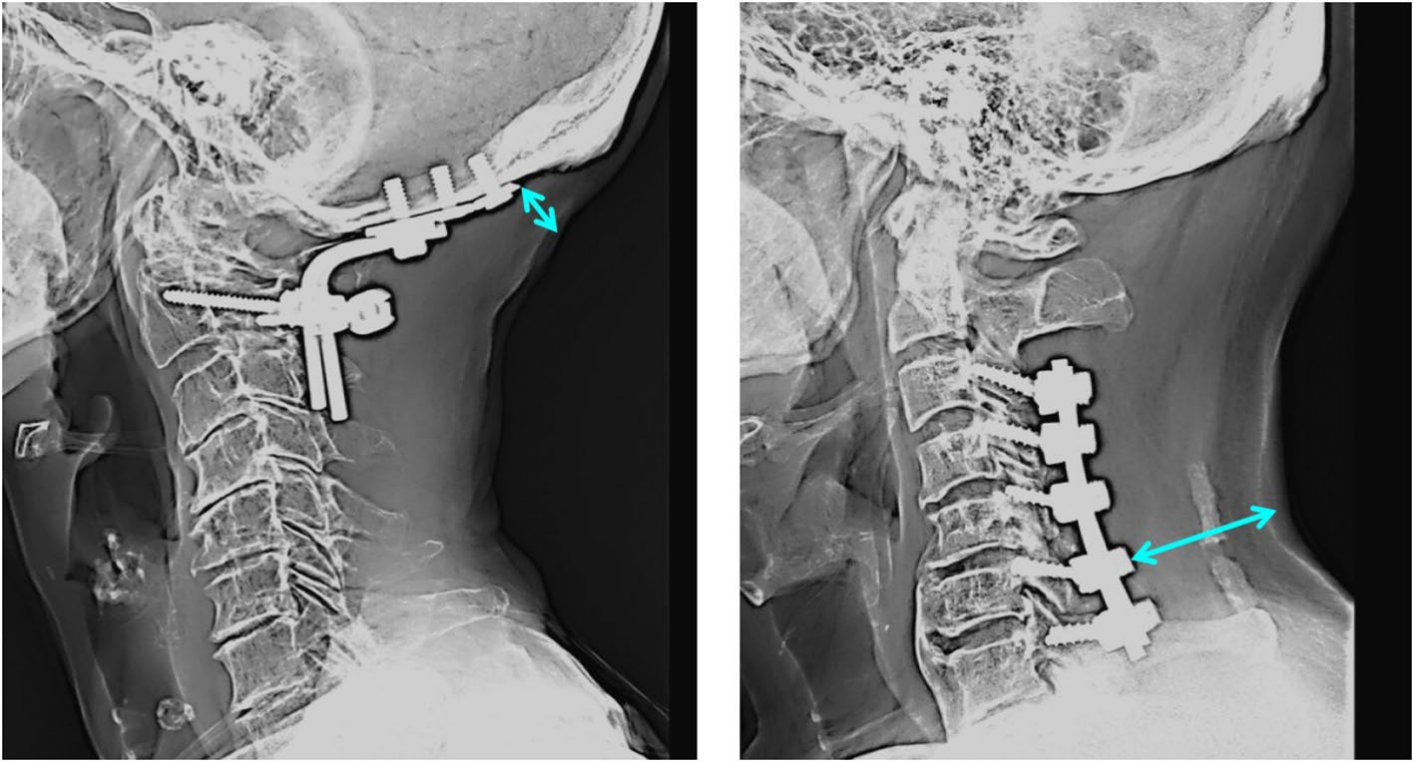
Left: Radiographic lateral image following occipito-C2 posterior instrumented fusion surgery. The soft tissues covering the metal implants placed on the occipital bone are relatively thin (bi-directional arrow). Right: Radiographic lateral image following sub-axial cervical spinal posterior instrumented fusion surgery. The soft tissues covering the metal implants are relatively thick (bi-directional arrow).

**Figure 2:**
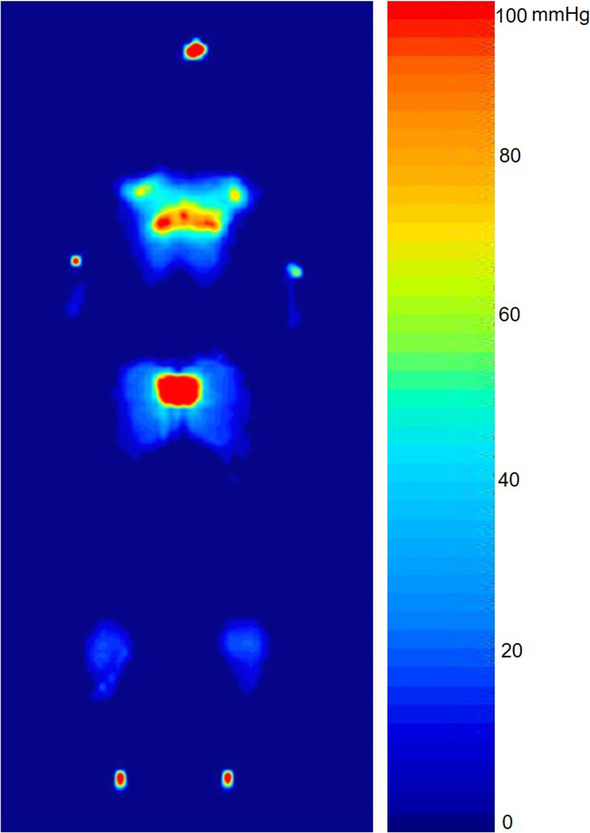
An example of a pressure image acquired from a male subject (weight 70 kg, height 170 cm) who was in the supine position without cervical orthosis. Note the increase in body interface pressure at the anatomical sites of the occipital area compared with the posterior side of the neck. Red areas indicate interface pressures of 90–100 mm Hg.

Occipitocervical surgery is a relatively rare procedure, and our findings were derived from a large sample size following a multicentre study design. To the best of our knowledge, this is the first study to identify occipitocervical procedure as an independent risk variable for SSI after posterior cervical spine surgery. We inferred the possibility of several factors adversely affecting the occurrence of SSI in patients undergoing posterior occipitocervical surgeries. First, the soft tissues covering metal implants placed on the occipital bones of patients who underwent occipitocervical instrumented posterior fusion were relatively thin compared with those covering the metal implants placed on the cervical spine (Fig. 1). Second, it is known that the body interface pressure at the occipital area increases significantly when an individual is in the supine position^28^ (Fig. 2), and it is also well known that the occipital area is an anatomical site at which pressure ulcers often occur, especially in patients who have experienced spinal cord injury^29,30^. On the other hand, mechanical compression of a surgical wound located posteriorly in the neck is unlikely to occur while the individual is in the supine position because the soft tissues are relatively thick and because of a lordotic curve in the cervical spine (Figs. 1 and 2). Third, the density of bacterial counts is reported to be particularly high in the scalp compared with that on the surface of the dorsal side of the trunk^31^. Furthermore, the cervical spine pathologies indicated for occipitocervical surgery are etiologically complicated in many cases and include rheumatoid arthritis, cancer, congenital cervical abnormalities, and spinal trauma^23–26^. These conditions may adversely affect the occurrence of SSI in posterior occipitocervical surgeries. Careful preoperative disinfection of the occipital area, atraumatic handling of the soft tissues covering the metal implant on the occipital bone during surgery, meticulous surgical wound closure, and alleviating pressure on any surgical wound in the occipital area when the patient is lying on a bed may help prevent SSI after posterior occipitocervical surgery. Future research ought to focus on the role that occipitocervical surgery may play in the pathogenesis of SSI in the spine.

Our multivariate analysis showed that male sex was significantly correlated with a higher incidence of deep SSI in the total cohort, although it was not a significant factor in univariate analysis. A limited number of studies to date have identified male sex as a significant independent risk variable for SSI after spine surgery^5–7^, although this relationship has been observed in several reports regarding trauma surgery, total joint arthroplasty, and abdominal surgery^32–35^. Neidhart et al. reported that male patients scheduled for orthopaedic surgeries were significantly more likely to carry nasal *Staphylococcus aureus* than were their female counterparts^36^.

Our present study had several limitations. First, the number of patients with SSI was relatively small (n = 28), as only patients who developed deep SSI after specific types of surgeries (posterior cervical spine procedure) were included; this contrasts with previous studies of SSI that generally focused on a wide variety of spine surgeries and all types of infections^2,9^. Patients’ registration, in the present research project, was finalized in June 2015, and we could not enrol additional patients. Therefore, since the number of event occurrence was relatively small (n = 28), there is a concern that potential risk related variables for deep SSI could not be detected as independent factors completely in the current analysis based on the clinical data collected during the 5 years from July 2010. Second, the study did not investigate several factors such as malnutrition^16^, plasma HbA1c levels^37^, and the surgeons’ level of experience^9^ that were reported as risk-related variables for SSI in previous literature. The results may still be biased due to unmeasured confounders, although certain confounding factors were adjusted when using multivariable analysis. In addition, several reports have described that the use of cervical orthoses tends to increase pressure ulcers around the neck, especially in patients who have suffered spinal cord injury^38–40^. In this multicentre observational study, information on the status of the postoperative use of cervical orthoses was not included in the clinical data collection, and the impact of postoperative use of cervical orthoses on the incidence of SSI following posterior cervical spinal surgeries could be a subject of further research. The strengths of the current research are the relatively large number of operative procedures covered and the multicentre observational cohort study investigating detailed potential risk variables for SSI following spine surgery utilising multivariable logistic regression analysis.

In conclusion, we found that occipitocervical surgery and male sex were independent risk variables for deep SSI after posterior cervical spine surgery in adult patients. These results may contribute to the surgeons’ awareness of the risk of SSI and allow for risk stratification as well as for patient counselling on the risks associated with posterior cervical spinal surgery. Furthermore, our findings could be helpful for developing protocols to decrease SSI risk in the future.
